# Supplementary material for: The Antidepressant Effect of Resveratrol Is Related to Neuroplasticity Mediated by the ELAVL4-Bdnf mRNA Pathway
Source: Int J Mol Sci. 2025 Jan 27;26(3):1113. doi: 10.3390/ijms26031113 (PMC11817429; doi:10.3390/ijms26031113)
Supplement: Supplementary file 1 [file ijms-26-01113-s001.zip › ijms-3393624-supplementary.pdf]

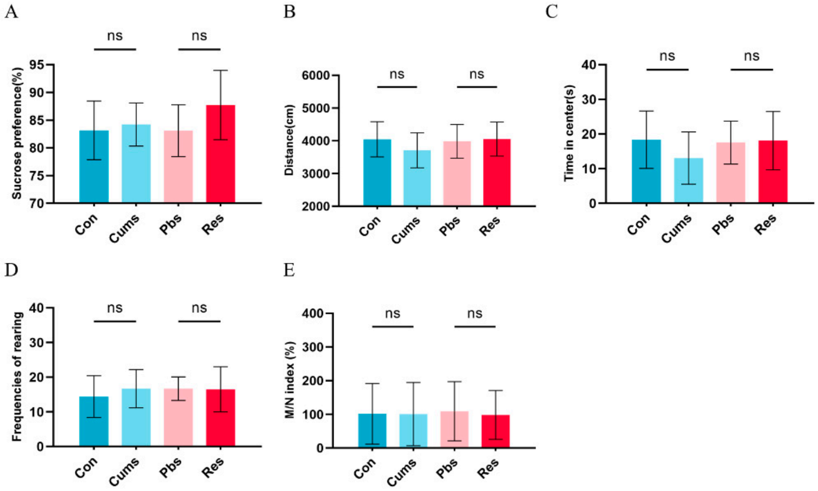

Figure S1. Baseline behavior tests (N = 10): (**A**) Preference index of SIT. (**B–D**) The total distance, time in center, and frequencies of rearing in OFT. (**E**) M/N index of the second phase of NOR. Statistical analyses are conducted using one-way ANOVA with Tukey's post hoc test (ns: no statistical significance).

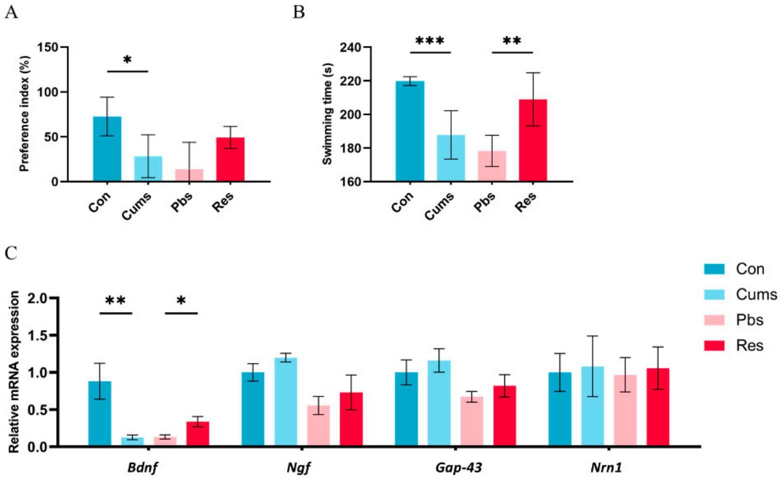

Figure S2. Supplementary behavior test (N = 6): (**A**) Preference index of SIT. (**B**) Swimming time of FST. (**C**) Relative *Bdnf*, *Ngf*, *Gap-43*, *Nrn1*/*Gapdh* mRNA expression. Statistical analyses are conducted using one-way and two-way ANOVA with Tukey's post hoc test ($^{*}p < 0.05$, $^{**}p < 0.01$, and $^{***}p < 0.001$).

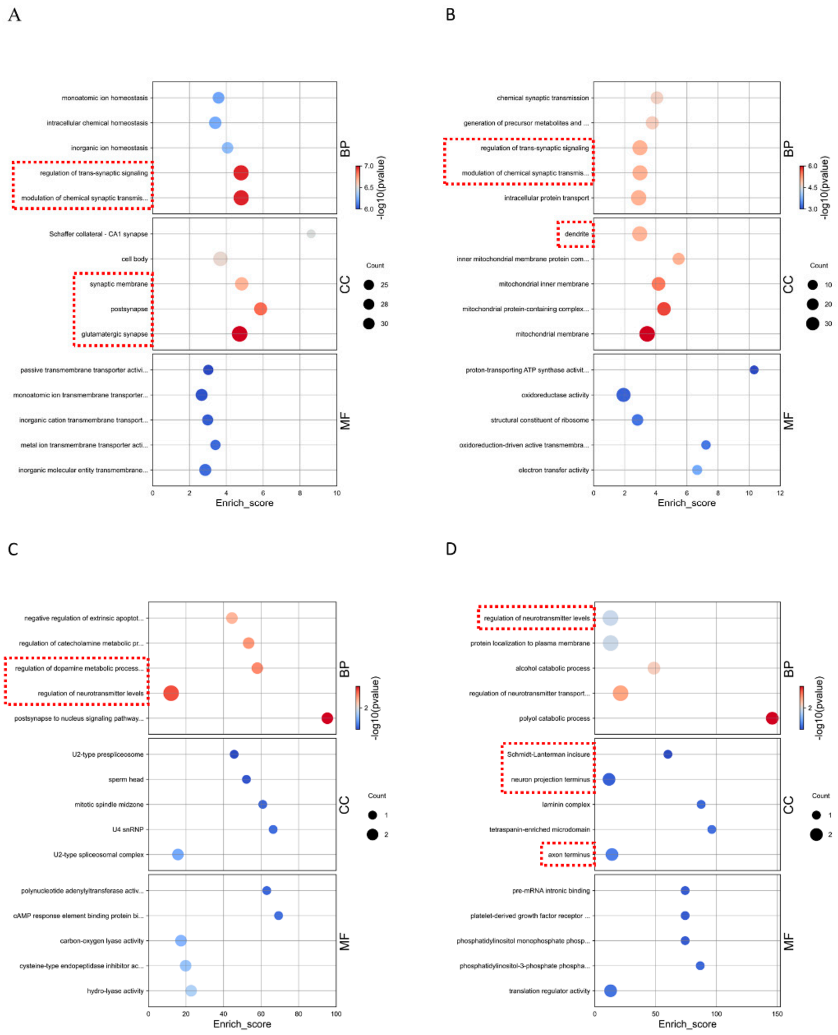

Figure S3. Resveratrol enhanced hippocampal neuroplasticity in rats: (**A–D**) Gene Ontology (GO) enrichment analysis of downregulated DEPs (Cums vs. Con) (**A**), upregulated DEPs (Cums vs. Con) (**B**), downregulated DEPs (Res vs. Pbs) (**C**), upregulated DEPs (Res vs. Pbs) (**D**). The red dotted box highlights changes associated with neuroplasticity.

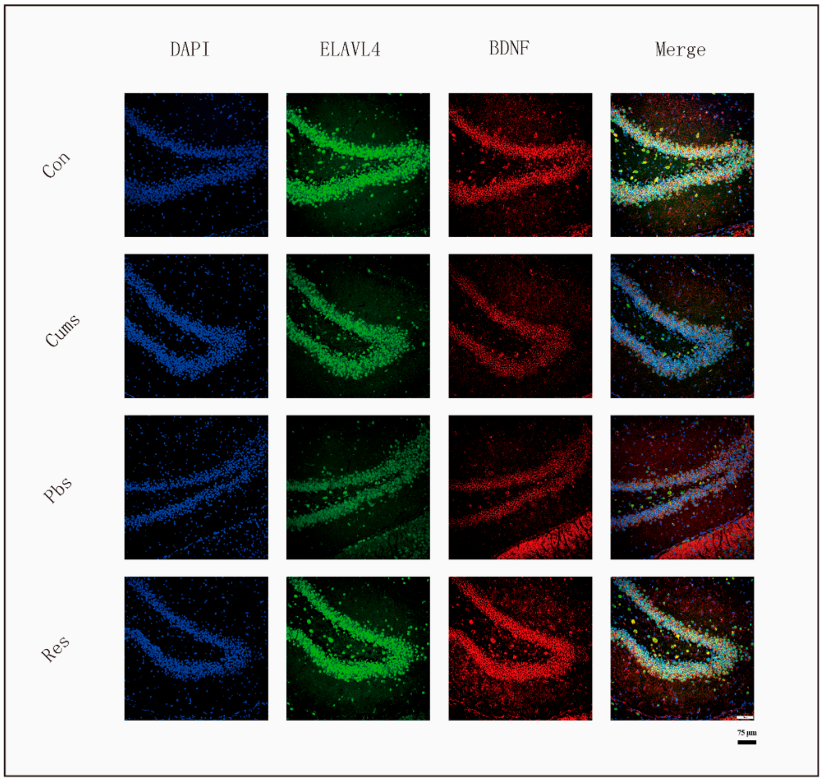

Figure S4. Representative immunofluorescence images showing ELAVL4 and BDNF expression in the DG region of the hippocampus (N = 3). Scale bars represent 75 μm.

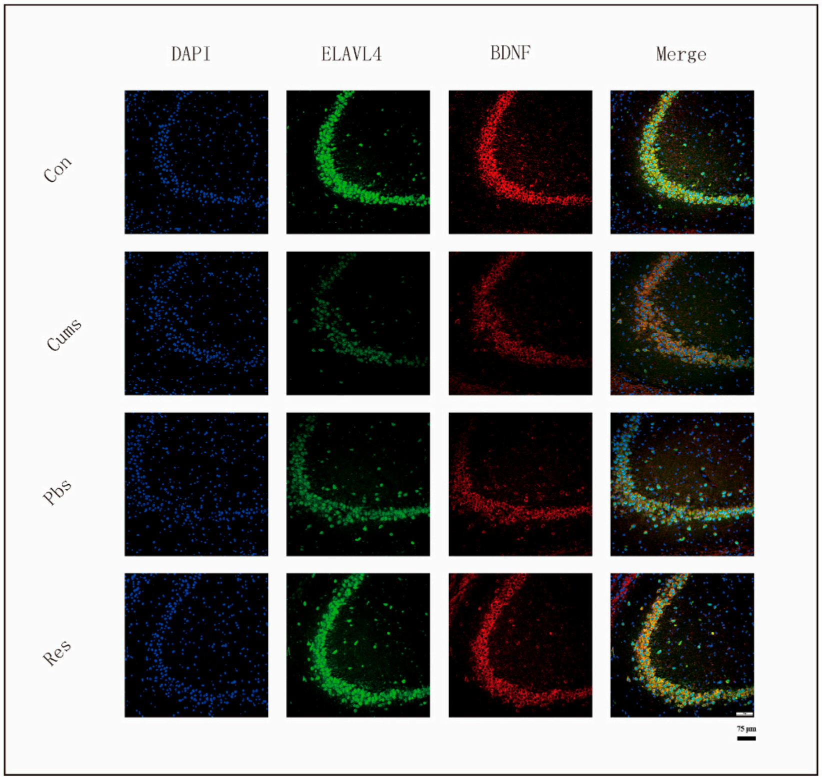

Figure S5. Representative immunofluorescence images showing ELAVL4 and BDNF expression in the CA3 region of the hippocampus (N = 3). Scale bars represent 75 μm.

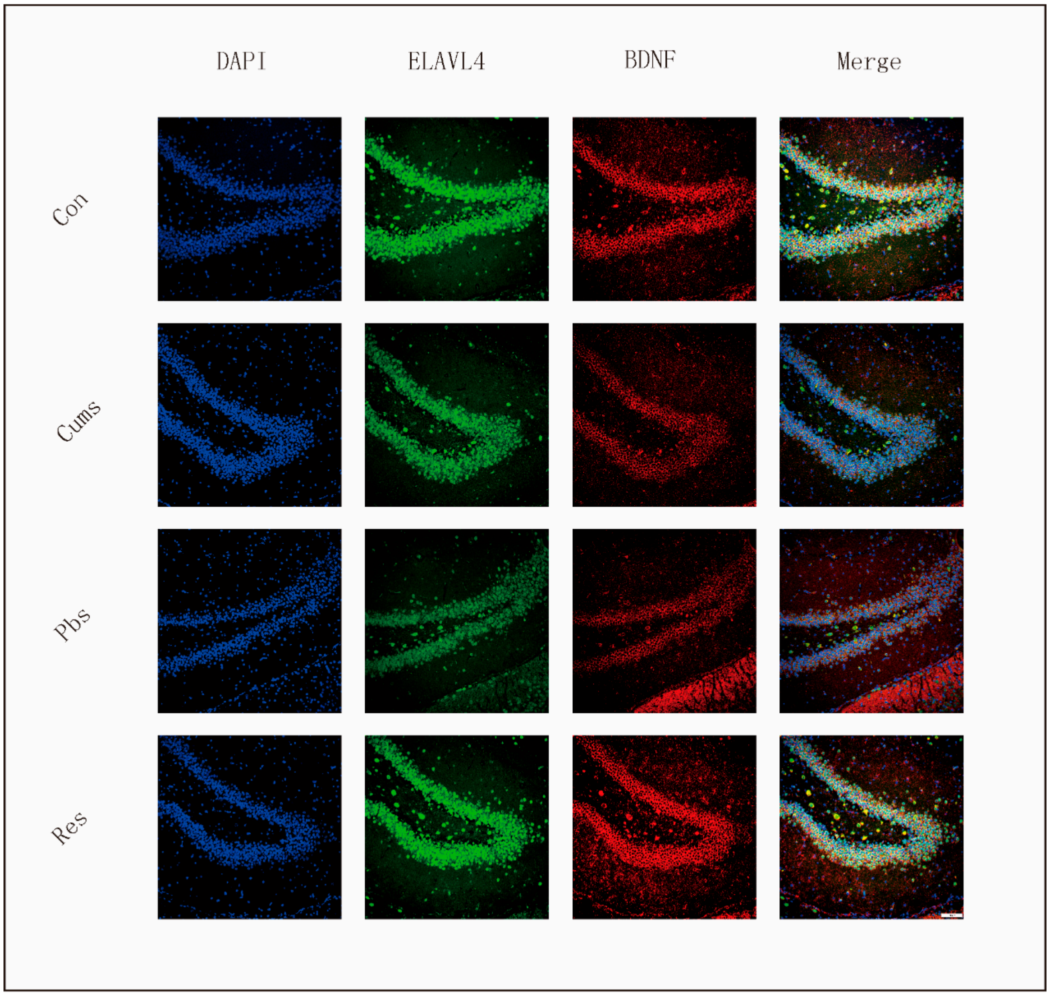

Figure S6. Representative immunofluorescence images showing ELAVL4 and BDNF expression in the DG region of the hippocampus (N = 3). Scale bars represent 75 μm.

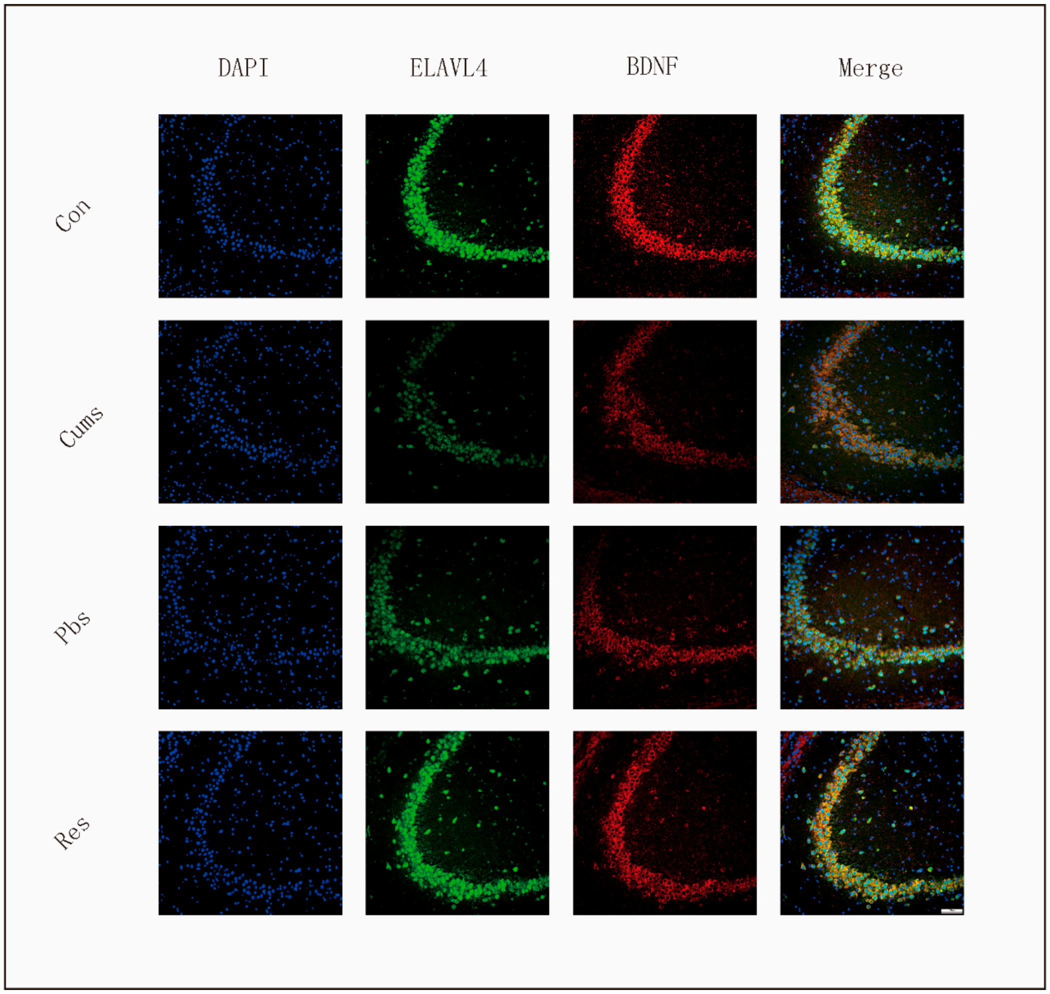

Figure S7. Representative immunofluorescence images showing ELAVL4 and BDNF expression in the CA3 region of the hippocampus (N = 3). Scale bars represent 75 μm.

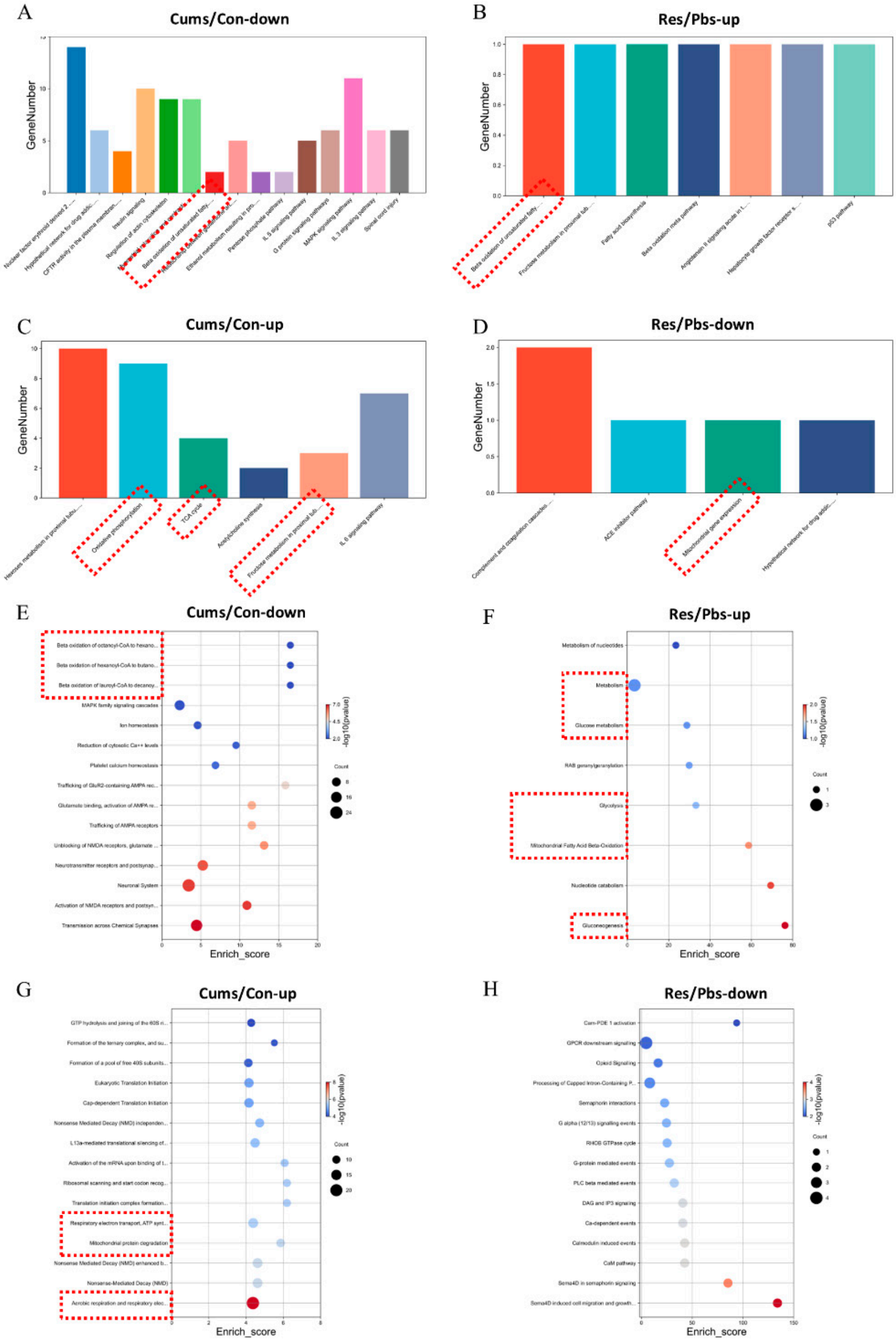

Figure S8. Supplementary results of proteomic analysis: (**A–D**) WikiPathways enrichment analysis of downregulated DEPs (Cums vs. Con), upregulated DEPs (Res vs. Pbs), upregulated DEPs (Cums vs. Con), and downregulated DEPs (Res vs. Pbs). (**E–H**) Reactome enrichment analysis of downregulated DEPs (Cums vs. Con), upregulated DEPs (Res vs. Pbs), upregulated DEPs (Cums vs. Con), and downregulated DEPs (Res vs. Pbs).

A

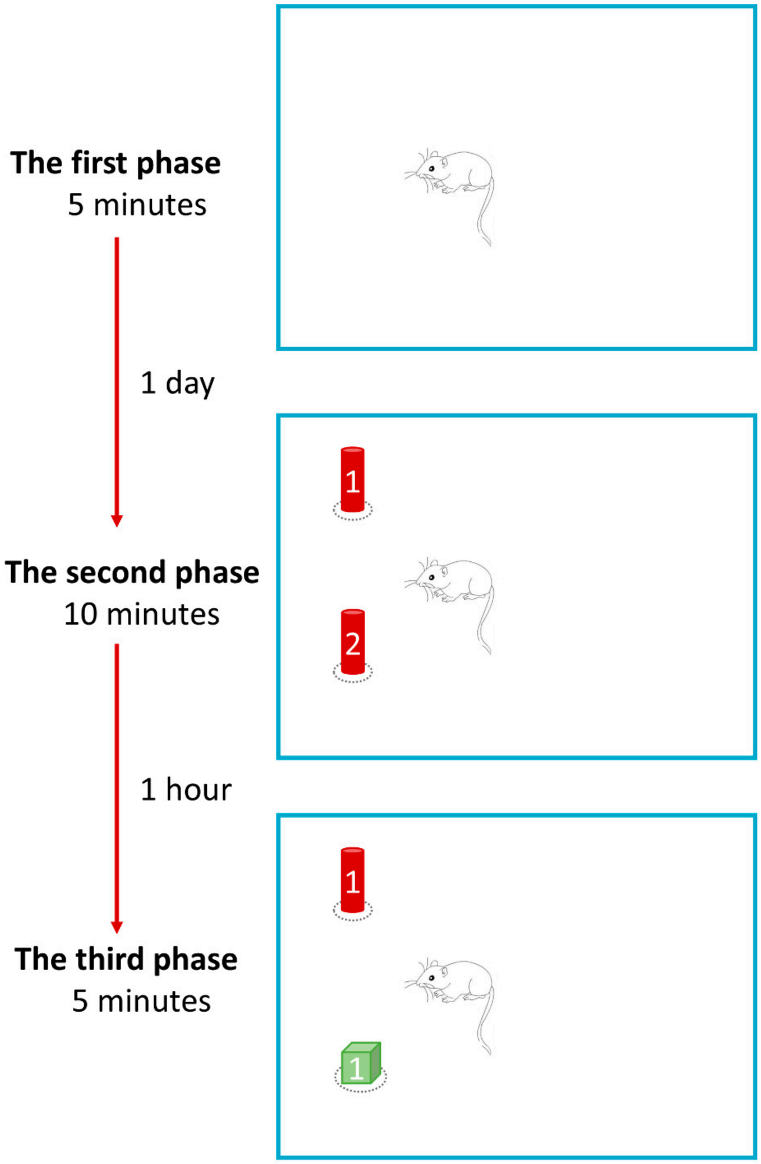

Figure S9. The NOR flow chart: (**A**) The NOR flow chart in this study.

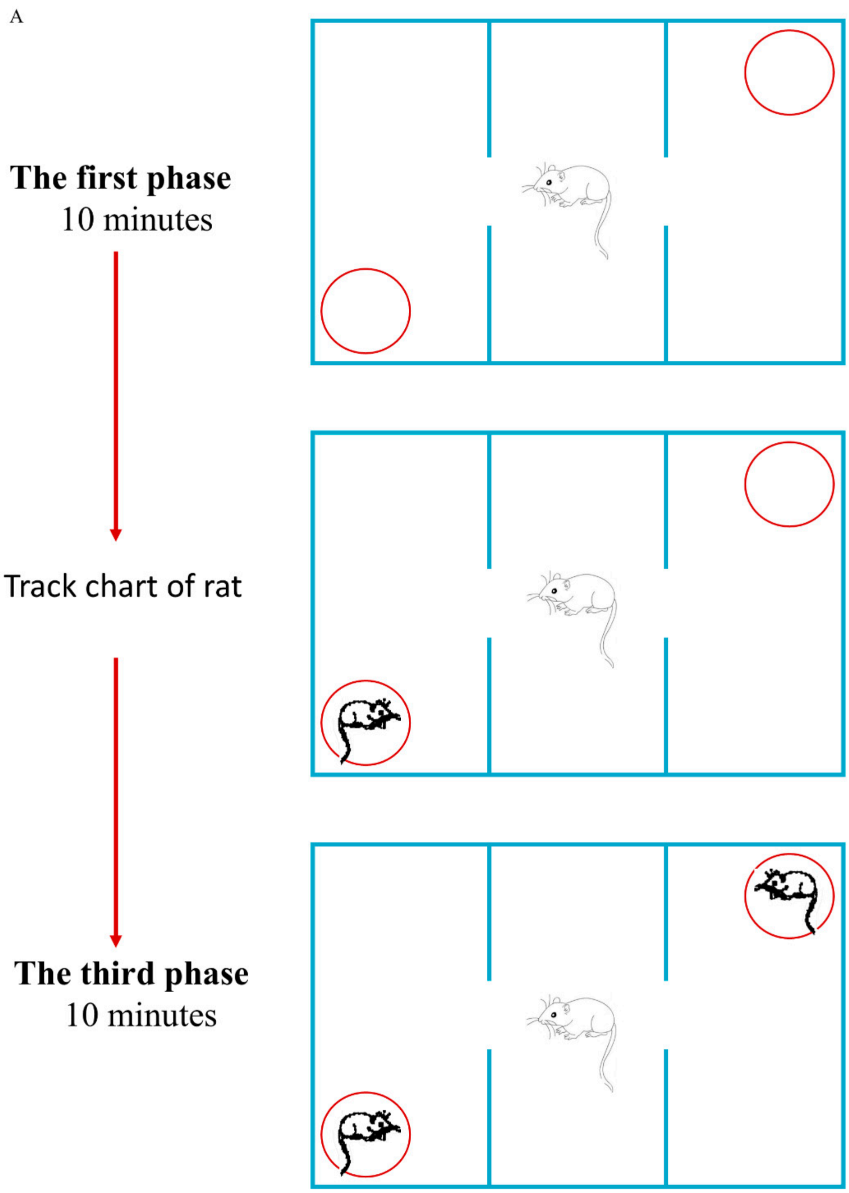

Figure S10. The SIT flow chart: (**A**) The SIT flow chart in this study.
